# Strategies for prevention of prolonged intensive care unit stay following cardiac surgery by identifying the determinants

**DOI:** 10.1186/1749-8090-10-S1-A165

**Published:** 2015-12-16

**Authors:** Philemon Gukop, Oswaldo Valencia, Mazin Sarsam, Vankatachallam Chandrasekaran, Brendan Madden

**Affiliations:** 1 Department of Cardiothoracic Surgery, St George's University Hospital NHS Foundation Trust, London, SW17 0QT, UK; 2 Department of Cardiorespiratory Medicine and Intensive Care, St George's University Hospital NHS Foundation Trust, London, SW17 0QT, UK

## Background/Introduction

Cardiac surgery service is dependent on the availability of cardiac intensive care facility. some patients are eligible for fast-track protocol. We investigate the factors determining prolonged intensive care stay following cardiac surgery, with the view to developing a model that predicts prolonged stay.

## Aims/Objectives

Develop a scoring model that predicts prolonged intensive care stay following cardiac surgery

## Method

Retrospective data analysis on 1592 consecutive patients admitted to intensive care following cardiac surgery (2011-2014). Dichotomous and categorical data were compared using Chi-square or Fisher's Exact tests. P- value of < 0.05 was significant. Univariate and Multivariate Regression identified predictors of prolonged intensive care stay.

A score model for prolonged intensive care stay was developed as a logistic probability unit (z=logit (p)= log e (p/1-p); The area under the receiver curve (AUC) generated. The best cut-off point of the scoring model was identified, the likelihood ratio of a positive test result calculated.

## Results

Logistic regression showed predictors of prolonged intensive care unit stay as ; NYHA class 3-4 (OR,1.5; p = 0.0029), FEV1 (OR, 0.76; p = 0.0026), emergency operation (OR,8.75; p = 0.0022), age (OR,1.02; p = 0.00007), LVEF<50% (OR,2.21; p = 0.00001), creatinine (OR,1.01; p = 0.000001), bypass time (OR, 1.01; p = 0.000001).

Intensive care unit stay score was determined by logistic probability (AUC=0.76, 95% CI, 0.73;0.78, p = 0.00001) suggesting that a cut-off score of 35 predicts prolonged intensive care stay with a sensitivity of 0.66, specificity of 0.72 and accuracy of 0.70. The likelihood ratio of a positive test was 2.34.

## Discussion/Conclusion

Preoperative optimisation of the predictors of prolong intensive care stay, could reduce length of stay following cardiac-surgery.

